# Elusive Gains of Cognitive Training: Limited Effects on Neural Activity Across Sessions

**DOI:** 10.3390/brainsci15010022

**Published:** 2024-12-29

**Authors:** Luka Juras, Andrea Vranic, Ivana Hromatko

**Affiliations:** Department of Psychology, Faculty of Humanities and Social Sciences, University of Zagreb, 10000 Zagreb, Croatia; ljuras@m.ffzg.hr (L.J.); avranic@ffzg.unizg.hr (A.V.)

**Keywords:** cognitive training, transfer of gains, qEEG, alpha power, theta power, coherence, temporal dynamics

## Abstract

Background/Objectives: Cognitive training paradigms rely on the idea that consistent practice can drive neural plasticity, improving not only connectivity within critical brain networks, but also ultimately result in overall enhancement of trained cognitive functions, irrespective of the specific task. Here we opted to investigate the temporal dynamics of neural activity and cognitive performance during a structured cognitive training program. Methods: A group of 20 middle-aged participants completed 20 training sessions over 10 weeks. Quantitative EEG (qEEG) parameters, including alpha and theta power, alpha/theta ratio, and fronto-parietal coherence, were analyzed at four time points to assess changes in neural activity. Results: Results revealed significant overall improvements in the trained task (n-back) performance, without an effect on the untrained task (OSPAN). qEEG analyses showed increased change in posterior (and a less robust in frontal) alpha power, particularly during mid-training, suggesting an improved neural efficiency in regions associated with attentional allocation and task engagement. Theta power remained stable across sessions, indicating a limited influence on neural processes underlying working memory and attentional control. The parietal alpha/theta ratio showed weak increases during mid-training, reflecting subtle shifts in the neural efficacy and cognitive engagement. There were no significant changes in functional connectivity between frontal and parietal locations. Conclusions: Our findings suggest that cognitive training primarily influences localized neural activity, rather than network-level connectivity. This lack of a longer-range network-level effect might also explain the failure of cognitive training paradigms to induce performance enhancements on the untrained tasks.

## 1. Introduction

Working memory (WM) refers to a set of cognitive processes responsible for temporarily holding and managing information necessary for an ongoing task [1]. It plays a crucial role in everyday life, enabling us to keep track of a conversation while formulating a response or remembering the goal of an activity. However, WM has inherent limitations: its capacity is finite, restricting the amount of information that can be processed simultaneously. These constraints affect the ability to efficiently manage complex tasks and are further exacerbated by age-related decline [2]. This decline is not exclusive to older adults; middle-aged individuals experience measurable reductions in WM performance, with a decrease of approximately one standard point between the ages of 50 and 69 [3].

In response to these challenges, numerous interventions have been proposed to enhance WM, with WM training emerging as a significant research area. WM training involves the repeated, standardized practice of WM tasks with the aim of improving WM and other related cognitive functions. Studies suggesting that gains from WM training might extend to broader abilities, such as fluid reasoning [4], have fueled considerable interest in such interventions. This enthusiasm is bolstered by the accessibility, low cost, and absence of adverse side effects associated with WM training, making it appealing to both scientific and mainstream audiences. However, the initial optimism surrounding WM training has been tempered by a growing skepticism. Concerns have been raised about exaggerated claims made by the brain training industry, often linked to tasks closely resembling those included in the training regimens [5]. Nearly two decades of research have yet to provide a definitive answer to the critical question: do training effects transfer to untrained tasks and a broader set of cognitive abilities? Meta-analyses conducted in the field reflect this ongoing debate. While some of them find evidence of broad transfer effects [6], others suggest that gains are largely confined to tasks that closely resemble the training exercises [7,8]. Even within the same cognitive domain, improvements often appear limited to structurally similar tasks [9], leaving the broader efficacy of WM training an open question.

Another key challenge in cognitive training research is understanding the mechanisms underlying the transfer to untrained tasks. Current models of transfer suggest that practicing a specific task can improve not only task-specific skills, but also broader domain-general cognitive mechanisms [10]. For instance, improvements in capacity constraints following WM training have been proposed as a mechanism driving improvements in fluid reasoning [4]. On a neural level, transfer is more likely when trained and untrained tasks involve overlapping neural substrates. In a landmark study by Dahlin et al. [11], the transfer effect was linked to increased activity in the striatal region activated during both the training and transfer tasks, even before the training began. In contrast, no transfer was observed for tasks that did not involve striatal activation. Moreover, age-related changes in the striatum were found to limit the extent of transfer. These findings highlight the importance of investigating neural changes as a function of training to understand specific neural adaptations and their contribution to task performance and (lack of) transfer to untrained tasks.

The studies that searched for biomarkers of change in neural efficacy as a function of training found some evidence of structural (e.g., [12,13,14]) and functional (e.g., [15,16,17,18,19,20]) change. Connectivity patterns between specific brain regions offer insights into the neural networks supporting cognitive improvements. However, most prior research adopts a pre-post training design, focusing on outcomes after training completion, while neglecting the dynamics of neural changes occurring during the training process itself. Previous studies suggest that increased connectivity between frontal and parietal regions facilitates attentional allocation and integration of sensory information. The systematization and consolidation of such findings, and the investigation of the relation between coherence indices and changes in the pattern of behavioral and cognitive scores, contribute to the integration of continuous qEEG measures as markers (of WM load) in adaptive cognitive trainings [21].

This gap leaves critical questions unanswered. How does neural activity evolve over the course of the training? What patterns of connectivity support these changes? And how do these dynamics relate to cognitive gains?

To address these questions, the current study investigates the temporal evolution of qEEG parameters across multiple stages of cognitive training. We explored how alpha, theta and their ratio change as participants progress through training. These two frequency bands were of interest as they have reliably been shown to correlate with performance in various cognitive domains [22,23,24]. While alpha-related metrics have generally been shown to positively correlate with memory and attention [25,26,27], theta power has been implicated in cognitive deficits in both healthy [28,29] and clinical [30,31,32] populations. Furthermore, their ratio has been identified as a potential indicator of cognitive ability in older adults [33,34]. We also analyzed the connectivity between frontal and parietal regions, under the assumption that cognitive training boosts synchronous coactivation of these circuits. We opted to clarify if and how network-level communication evolves to support learning and cognitive transfer. To that aim, we used both trained (n-back) and an untrained (OSPAN, i.e., Operation Span) task to test for the transfer of training gains. In an n-back tasks, participants compared the current stimulus with one presented *n* steps earlier in the sequence. As the value of *n* increased, the task became more challenging, placing greater demands on WM. Similarly, OSPAN task required participants to alternate between solving simple equations and memorizing presented letters. This dual-task structure tests the ability to update WM, shift between subtasks, and suppress irrelevant information. However, success in both tasks depends on recalling items in the correct sequence, which involves several critical processes [35]. These include forming strong associations between content (presented stimulus) and context (position), preserving these associations while handling interference from new stimuli or a secondary task, and efficiently updating associations between trials. Studies comparing the qEEG patterns between various WM tasks, partly confirm their hypothesized conceptual commonalities [36].

Based on these findings and theoretical expectation that cognitive training facilitates neural plasticity, we hypothesized that an initial increase in theta activity, reflecting heightened attentional engagement, shall appear in early sessions, followed by a decline as participants achieve greater task-solving efficiency. Conversely, we expected alpha activity to increase over time, indicating improved task automation. We also expected enhanced connectivity between frontal and parietal regions to appear as a function of the training, aligning with improved coordination between attentional control and sensory integration networks. Finally, we hypothesized that the transfer of gains to the untrained task will be mediated by these modulations of qEEG activity as a function of cognitive training.

Furthermore, our study focuses on late middle-aged adults, a population at a critical juncture in cognitive aging. The potential effects of cognitive training might prove particularly relevant for this age group, as it may bolster neural resilience and slow age-related decline in cognitive abilities.

## 2. Materials and Methods

### 2.1. Participants

Out of a larger sample of participants undergoing cognitive trainings, a subsample of 20 adults aged 47 to 65 (M = 55.1, SD = 4.01) participated in the EEG recordings conducted during four cognitive training sessions. The sample included 13 women, and eight participants had a high school diploma, two held a bachelor’s degree, nine had a master’s degree, and one participant held a PhD. Participants were recruited through collaborations with workplace organizations and personal contacts. Inclusion criteria required participants to report no history of: (1) psychiatric or neurological disorders; (2) use of anti-dementia or psychotropic medication; (3) significant visual or hearing impairments; or (4) other conditions that could limit their ability to work. For their participation, they were symbolically rewarded with a 15 EUR voucher for a local drugstore.

### 2.2. Procedure

**Figure 1:**
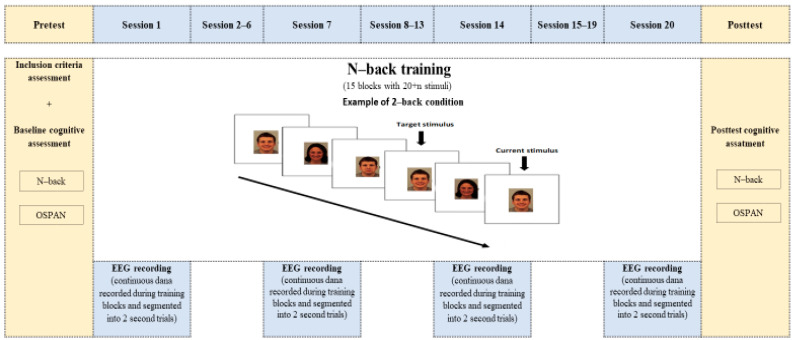
Schematic presentation of study design. Participants completed 20 training sessions and EEG data were recorded during the 1st, 7th. 14th and 20th sessions.

Before the training, participants were informed that the study aimed to investigate the effects of various activities on cognitive abilities. Participants completed WM tasks prior to and immediately following the training sessions. EEG data were collected during the 1st, 7th, 14th, and 20th training sessions in the non-shielded environments, either at the participants’ homes or workplaces (see Figure 1 for a schematic presentation of study design). The study was approved by the Ethical Committee of the research institution, and all participants provided written informed consent in accordance with the Declaration of Helsinki.

N-back [37]. In this task, participants were required to press a designated key whenever the currently presented stimulus matches the *n* stimuli shown earlier. The task included two blocks for each of the 1-back, 2-back, and 3-back difficulty levels. Each block consisted of 20-*n* stimuli, featuring eight photographs selected from the Karolinska Directed Emotional Faces database (KDEF; [38]). The photographs depicted one male and one female model, each displaying one of four basic emotions: sadness, happiness, anger, and surprise. Before starting the main task, participants completed a practice block for each difficulty level to familiarize themselves with the procedure. The total task score was calculated as the proportion of correctly recognized stimuli (hits) minus the proportion of false alarms. 

Operation Span Task (OSPAN; [39]). In this task, participants were required to remember a sequence of letters, ranging from 3 to 7 in length, each displayed for 1 s. After each letter, participants solved a short algebra task involving two operations (e.g., addition and subtraction). Following the completion of a sequence, participants were asked to recall the shown letters in the correct order. Each sequence length was presented three times. Before the main task, participants completed practice trials to familiarize themselves with the procedure. The OSPAN score was calculated based on the number of letters recalled in the correct order.

### 2.3. Training

Participants completed the self-administered training sessions twice a week for 10 weeks, totaling to 20 sessions. Each session lasted approximately 20 min and was accessed on participants’ personal computers. The training consisted of an adaptive n-back task with 15 blocks per session, allowing for adjustments based on individual performance both within and between sessions. Participants began training at the 1-back level of difficulty. If a participant made two or fewer mistakes in an n-back block, they progressed to the next level. Conversely, if they made five or more mistakes, the following block was reduced by one level of difficulty. Participants earned points for each completed block, with point values reflecting the difficulty level. At the end of each session, they received virtual medals based on their highest level of achievement.

### 2.4. EEG Data Recording and Preprocessing

EEG data were recorded using a Mobita 32-Channel Wireless EEG System (Biopac Systems Inc., Goleta, CA, USA), with electrodes placed according to the international 10/20 extended system: Fp1, Fpz, Fp2, F7, F3, Fz, F4, F8, FC5, FC1, FC2, FC6, T7, C3, Cz, C4, T8, TP9, CP5, CP1, CP2, CP6, TP10, P7, P3, Pz, P4, P8, PO, O1, Oz, and O2. EEG was recorded during the training sessions. However, due to technical issues, EEG recordings for three participants, at certain time points are missing. For the analysis, electrodes were grouped by region: frontal electrodes included Fp1, Fp2, F7, F3, Fz, F4, and F8, while parietal electrodes included Pz, P3, P4, P7, P8, and PoZ. EEG data were preprocessed and analyzed using MATLAB (Version R2020b) and the FieldTrip toolbox [40].

EEG data were recorded at a sampling rate of 1000 Hz and were not down-sampled for subsequent analyses. A high-pass FIR filter with a cutoff frequency of 1 Hz and a low-pass FIR filter with a cutoff frequency of 40 Hz were applied, both with the filter order and transition band automatically determined by FieldTrip’s default settings. Additionally, a notch filter targeting the 50 Hz frequency was used to remove line noise. The data were re-referenced to the average of all electrodes, including the initial reference used by the Mobita system, to preserve the full rank of the data [41]. Data were recorded during each training block and segmented into non-overlapping 2-s epochs corresponding to the training performance periods. Time segments were visually inspected to remove those containing noisy data due to head movement, chewing, frequent or severe eye blinks, or electrode pops. Channels with persistent issues across multiple time segments were excluded from the analysis. After removing bad trials and electrodes, Independent Component Analysis (ICA) was performed using the *runica* algorithm implemented in the FieldTrip toolbox. The analysis included all channels, with data demeaned prior to computation, and dimensionality reduction was applied using Principal Component Analysis. Components related to eye movements, muscle activity, and other artifacts were manually identified by inspecting their topographies, time-series, and power spectra, and were subsequently removed. Finally, after ICA, time segments were manually inspected again, and any remaining segments with residual artifacts were removed.

### 2.5. Data Analysis

The multi-taper FFT (mmft) method was used to estimate the power spectral density (PSD) of EEG data, employing Hanning tapers. These tapers are particularly effective for spectral estimation, as they minimize spectral leakage, ensuring that power is accurately assigned to the appropriate frequency bins. The number of tapers was determined by the default settings in the FieldTrip toolbox. The time-bandwidth product was set to 1 Hz, which influences the smoothness of the frequency estimates. The frequency range of interest spanned from 1 to 30 Hz, a common range in EEG analysis that encompasses brain wave activity of interest int this study: Theta (4–7 Hz) and Alpha (8–13 Hz) waves. These bands were chosen because they are linked to specific cognitive and neural processes: theta waves are associated with attentional engagement, while alpha waves reflect performance automatization. For evaluation, PSD served as the primary measure to assess the distribution of power across different frequencies, offering insight into the brain’s oscillatory activity. Additionally, relative alpha and theta power were calculated to control for individual differences in skull thickness and volume conduction.

Coherence, a measure of the linear relationship between signals in the frequency domain, was calculated to assess functional connectivity between predefined electrode pairs across the 1–30 Hz frequency range. Coherence was computed independently for five electrode pairs: {‘F3’, ‘P3’}, {‘F4’, ‘P4’}, {‘Fz’, ‘Pz’}, {‘F3’, ‘P4’}, and {‘F4’, ‘P3’}. The analysis utilized the *ft_connectivityanalysis* function from the FieldTrip toolbox, with the method parameter set to *coh* for magnitude-squared coherence. Coherence values were obtained for each frequency bin within the 1–30 Hz range and then averaged across this frequency range to provide a summary measure of functional connectivity.

All statistical analyses were conducted in JASP (JASP team, [42]). To compare gains on n-back and OSPAN task, GLM was conducted for repeated measures. A repeated measures ANOVA was conducted to examine changes in frontal and posterior alpha power, theta power, and the alpha/theta power ratio across training sessions. To complement the ANOVA results, Bayes factors (BF10) were calculated as an alternative measure of evidence for the presence of effects. Specifically, BF10 was used to evaluate the strength of evidence for the alternative hypothesis (H1) compared to the null hypothesis (H0). The classification scheme by Lee and Wagenmakers [43] was employed to interpret the BF10 values. Values greater than 1 and less than 3 indicate “anecdotal” evidence for H1; values between 3 and 10 suggest “moderate” evidence; and values from 10 to 30 signify “strong” evidence for H1. Conversely, BF10 values lower than 1 indicate support for H0, with values between 0.33 and 1 denoting “anecdotal” support; BF10 values between 0.1 and 0.33 reflecting “moderate” evidence; and values from 0.01 to 0.1 suggesting “strong” evidence in favor of H0. The assumptions of sphericity were assessed using Mauchly’s test, with Greenhouse-Geisser corrections applied when violations occurred to adjust the degrees of freedom. Post-hoc comparisons were carried out using Bayesian analysis to evaluate differences between specific levels of the repeated measures factor, with corrections for multiple comparisons implemented according to the method described by Westfall et al. [44].

## 3. Results

**Figure 2:**
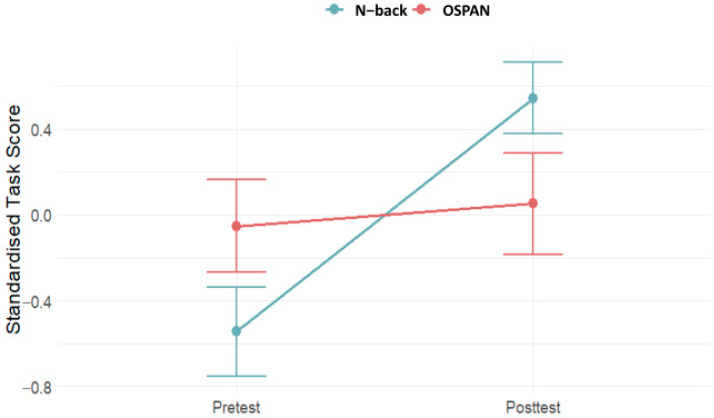
The average standardized task scores on n-back and OSPAN task at pretest and posttest (N = 20). Vertical lines represent the standard error.

Participants demonstrated an overall improvement in their performance throughout the training sessions (Appendix A). At the beginning of the training, participants exhibited lower variability in their performance. As the sessions progressed, they showed significant improvement from session to session. However, by the end of the 20 training sessions, the rate of improvement plateaued, and the variability in results increased (see Appendix A). This pattern suggests that while participants initially benefited from the training, the learning curve leveled off, resulting in greater fluctuations in performance among participants. Figure 2 presents average standardized task scores on n-back (trained) and OSPAN (untrained) task at pretest and posttest. A repeated measures GLM showed a significant improvement in post-training scores on the trained (n-back) task (F = 11.83, df = 1/19, *p* < 0.001.), and no changes in the performance on the untrained task (OSPAN; F = 0.25, df = 1/19, *p* = 0.622). 

**Figure 3:**
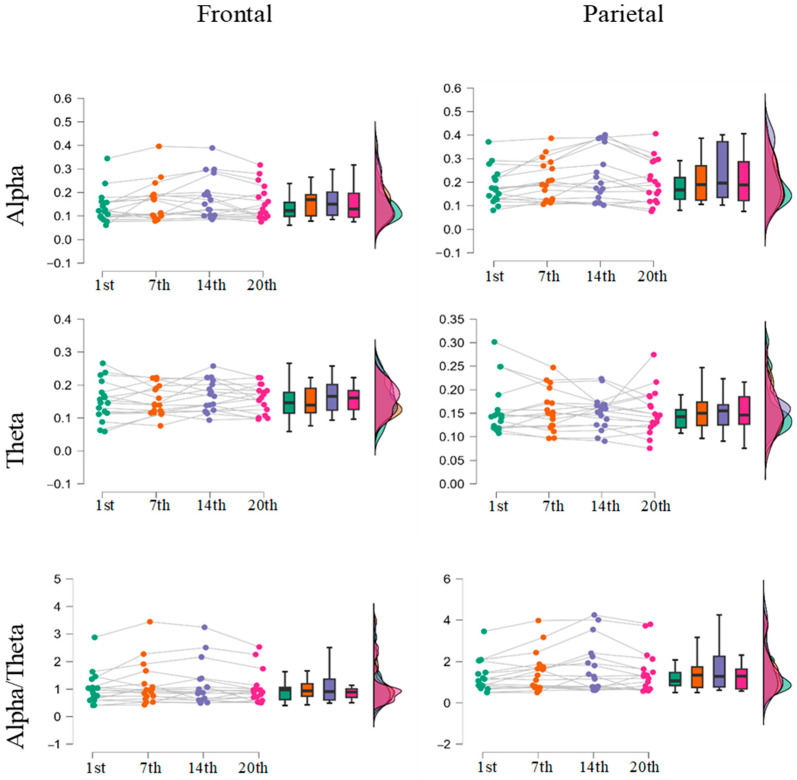
The frontal and parietal alpha/theta ratio, theta and alpha band power for all participants across the four measurement points (N = 20).

Figure 3 presents individual results for all participants, showcasing various qEEG power measures across the four measurement points. Notable individual differences were observed at each measurement point. The results of repeated measures ANOVA for alpha and theta power across training sessions is shown in Table 1.

### 3.1. Alpha Power

A repeated measures ANOVA revealed a significant effect of measurement points on frontal alpha power (*F*(3, 48) = 4.134, *p* = 0.01, *η*^2^ = 0.205, BF10 = 4.249). Post-hoc comparisons conducted using Bayesian analysis assessed the differences in alpha power between training sessions. Weak evidence indicated an increase in alpha power at the 7th (BF10 = 1.8) and 14th (BF10 = 2.8) training sessions compared to the 1st session. For the final session, anecdotal evidence suggested no significant differences in frontal alpha power between the first and last training sessions (BF10 = 0.879).

In contrast, very strong evidence was found for differences in posterior alpha power across training sessions (*F*(3, 48) = 7.692, *p* < 0.001, *η*^2^ = 0.325, BF10 = 96.597). Post-hoc comparisons revealed significant differences between the 1st session and subsequent sessions. Notably, the comparison between the 1st and 7th sessions indicated moderate evidence of an increase in posterior alpha power (BF10 = 3.31). Strong evidence was also found for an increase in parietal alpha power during the 14th session compared to the 1st session (BF10 = 14.83). However, the comparison between the 1st and 20th sessions provided only weak evidence for an increase in alpha power (BF10 = 1.88). Overall, the findings suggest that the greatest increase in alpha power occurred around the 14th session, although this increase diminished by the final session.

### 3.2. Theta Power

For both frontal and parietal theta power, substantial evidence supported the null hypothesis (BF < 0.33), indicating that theta power did not change throughout the training sessions and remained stable.

### 3.3. Alpha/Theta Power Ratio

Regarding the alpha/theta power ratio, a distinctive pattern emerged across frontal and parietal regions. Although the repeated measures ANOVA (Table 1) found no significant differences across training sessions, the Bayes factor provided weak support for the null hypothesis (BF10 = 0.612). However, substantial evidence indicated changes in the alpha/theta power ratio over parietal electrodes (*F*(3, 48) = 4.183, *p* = 0.010, η^2^ = 0.207, BF10 = 4.39). Post-hoc analysis revealed weak evidence for increases in the alpha/theta ratio at the 7th (BF10 = 2.343) and 14th (BF10 = 2.221) training sessions. No significant support was found for either the null or alternative hypotheses at the 20th training session (BF10 = 0.985).

### 3.4. Fronto-Parietal Coherence

The coherence analysis for the selected pairs of electrodes across the four recording sessions (1st, 7th, 14th, and 20th training sessions) showed no significant differences (see Table 2). The *F*-values were low for all electrode pairs (F3–P3, F3–P4, F4–P3, F4–P4, and Fz–Pz), with *p*-values above 0.05, indicating non-significant results. The Bayes factors (BF10) were close to 0.1, suggesting that the data provide strong evidence in favor of the null hypothesis, supporting the conclusion that there were no meaningful changes in coherence across sessions.

## 4. Discussion

The significant overall improvement in the trained task performance throughout the training sessions suggests that participants benefited from training, at least in the specific context of the trained task. However, the findings of no improvements in the untrained task does not support the notion of a boosting effect of cognitive training on cognitive abilities in general. Regarding the trained task (n-back), the observed plateau in the performance improvements by the 20th session reflects a common learning curve pattern where gains become less pronounced after initial stages of rapid improvement. This indicates that participants reached a performance ceiling for the task. Increased variability in the performance towards the end of training implies a strong effect of individual differences in cognitive capacities, motivation, or engagement as the training progressed. These findings align with established learning theories, which describe initial rapid improvements followed by stabilization as participants master the task [45], as well as cognitive training studies reporting an asymptotic pattern of change, whereby large cognitive and neural improvements observed in the early stages of the training intervention tend to plateau over time [46,47,48,49].

Alpha and theta power analysis. The weak evidence for increases in frontal alpha power across sessions suggests only marginal changes in cognitive states, which might indicate that the cognitive training program did not heavily engage frontal regions. The observed changes might be a mere by-product of the familiarity with the task. Strong evidence for increases in posterior alpha power, particularly around the 14th session, does suggests enhanced neural efficiency in regions linked to visual processing and attentional allocation, while the diminished changes by the 20th session could indicate either a stabilization of neural adaptations or a reduced demand on attentional processes as tasks became more automated. Given the adaptive nature of the n-back task, with increasing levels of item difficulty as the participant advances, this study cannot provide definite conclusions regarding this issue. Moreover, it should be noted that the shifts as a function of training were only observed with alpha frequency, while none of the other qEEG parameters showed significant changes, which does not provide substantial support for the claim that cognitive training resulted in any meaningful reorganization of neural activity. 

Indeed, the absence of changes in both frontal and parietal theta power suggests that cognitive training did not substantially influence neural processes linked to working memory, cognitive control, and encoding [22,50,51,52]. This stability may indicate that the cognitive training tasks were not sufficiently demanding to elicit substantial theta-related activity. However, based on the reached plateau, this does not seem likely. Alternatively, this sort of cognitive training might modulate attention allocation and the inhibition of irrelevant information [53,54], without exerting effects on WM capacity per se. This explanation would be in line with the finding that n-back tasks, used in this study, measure different capacities in younger vs. older participants. Performance was most related to executive functions in participants of young age, while a combination of attentional and executive processes was associated with performance in middle-aged participants [55].

Weak evidence for changes in the alpha/theta ratio over parietal regions indicates subtle shifts in neural efficacy around the 7th and 14th sessions, pointing to a period of heightened neural efficiency during mid-training. This increase in the parietal alpha/theta ratio aligns with improved task automation and reduced cognitive effort as training progressed. However, the lack of robust changes by the final session suggests that the benefits of training in terms of neural efficiency may be short-lived without continued engagement.

Coherence Analysis. The absence of significant changes in coherence across training sessions suggests that functional connectivity on the fronto-parietal plane remained stable throughout the training process. Bayes factors strongly favoring the null hypothesis confirm that cognitive training did not alter connectivity patterns in the regions analyzed. These findings may indicate that while cognitive training influenced localized activity (e.g., parietal alpha power), it did not drive network-level changes in connectivity between frontal and posterior regions, a putative biomarker of processes related cognitive control [18]. This could be due to the nature of the training tasks, which may not have required significant cross-regional communication. Additionally, the lack of such network-level effects might explain the lack of effect on an untrained task. Robust and meaningful increases in fronto-parietal connectivity were expected as a function of training, and as such were hypothesized to be the driving force underlying transfer of gains. As it is, the conducted n-back training does not provide enough evidence for the hypothesized conceptual overflow of training-related cognitive performance gains into the neural realm. 

Potential implications and directions for future research. The results of our study, when considered within the broader body of literature, highlight several important implications for future research. One key area for investigation is the strategies participants use during the WM training. While strategic training has been shown to improve performance on the trained task, these strategies often fail to transfer to untrained tasks [56]. Recent theoretical frameworks have differentiated between transfer effects arising from increased cognitive capacity and those stemming from improved cognitive efficiency [57]. Exploring factors related to cognitive efficiency could be particularly relevant when employing computer-based cognitive assessment and training protocols, especially among middle-aged and older adults. Indeed, digital exclusion remains an important issue, even in developed countries and among people with higher socioeconomic status [58]. This is supported by the reactions of some participants in our study, who spontaneously expressed concerns about making unintentional mistakes while using technology. As a result, training gains in these populations could be partially attributed to improvements in technological proficiency and reduced anxiety with digital tools. Furthermore, future research should move beyond focusing solely on cognitive factors and incorporate a broader range of variables as predictive or related to training performance. For example, some participants reported that the repetitive nature of the n-back task was not engaging, and they questioned how repeated practice could translate into improvements in overall cognitive function or everyday cognitive performance. Including more informative feedback and gamified motivational instances, both visual- (i.e., changes in color palette, background) or content-wise (i.e., different stimulus categories, scoreboard), as well as measures of motivation, both within and across trial bocks might be a direction to investigate this issue further [59,60]. 

## 5. Conclusions

In summary, our results highlight the alpha band neural activity changes in distinct phases during the training, with mid-training sessions (e.g., 7th and 14th) showing the most pronounced alterations. These findings suggest that mid-training may be a critical period for cognitive engagement and learning, warranting targeted interventions to maximize gains. However, the stability of theta power and coherence, as well as the absence of transfer to the untrained task, raise questions about the broader neural mechanisms underpinning training related improvements. Future studies could explore whether specific task designs or feedback mechanisms are necessary to elicit changes in these metrics. Some neurofeedback studies showed promising effects in that regard, while others reported supremacy of cognitive training over neurofeedback in boosting WM in healthy adult subjects [61]. Finally, the increasing variability in task performance underscores the importance of personalized cognitive training programs, as well as the need for further research into the neural mechanisms of cognitive training, particularly to understand how to sustain and extend its benefits.

## Figures and Tables

**Table 1:** Results of repeated measures ANOVA for alpha and theta power across four training sessions.

	F	df	p	η^2^	BF10
Frontal alpha	4.13	3/48	0.011	0.205	4.25
Parietal alpha	7.69	3/48	<0.001	0.325	96.60
Frontal theta	1.40	3/48	0.255	0.080	0.32
Parietal theta	0.28	3/48	0.840	0.017	0.11
Frontal alpha/theta	2.07	3/48	0.117	0.014	0.612
Parietal alpha/theta	4.18	3/48	0.010	0.207	4.39

**Table 2:** The results of repeated measures ANOVA for coherence between selected pairs of electrodes (*N* = 20).

Electrode Pairs	F	df	p	BF10
F3–P3	0.09	3/48	0.963	0.090
F3–P4	0.24	3/48	0.871	0.102
F4–P3	0.38	3/48	0.770	0.116
F4–P4	0.39	3/48	0.760	0.119
Fz–Pz	0.37	3/48	0.778	0.116

**Figure A1:**
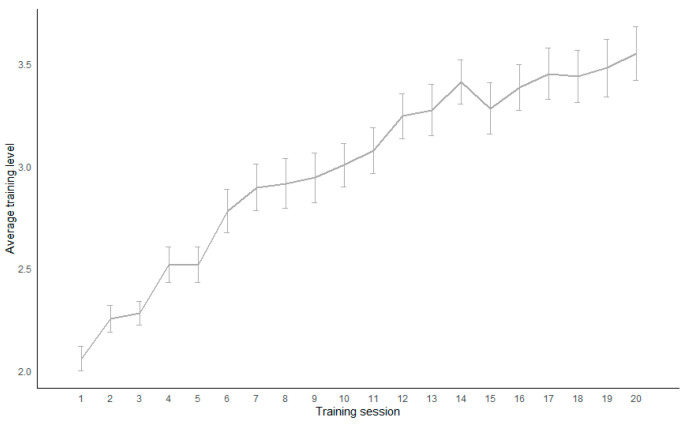
Training sessions. Vertical lines represent the standard error at each session.

## Data Availability

Because this is an ongoing study and the data is still being collected, the data that support the findings of this study are available from the first author, [L.J.], upon reasonable request.
